# Covalent anchoring of tethered ligands to chemogenetic handles for targeted *in vivo* neuropharmacology

**DOI:** 10.1016/j.xpro.2025.104186

**Published:** 2025-11-11

**Authors:** Maria Ciscato, Kian Stenbolt, Alexandre Mourot, Nicolas Guyon

**Affiliations:** 1 Brain Plasticity Unit, ESPCI Paris, PSL University, CNRS, 75005 Paris, France; 2 Department of Neuroscience, Karolinska Institutet, 171 77 Stockholm, Sweden

**Keywords:** Cell Biology, Microscopy, Molecular Biology, Neuroscience, Behavior, Molecular/Chemical Probes

## Abstract

Covalent anchoring of tethered ligands to chemogenetic handles (CATCH) is a chemogenetic approach that enables sustained, localized, and pharmacologically specific receptor antagonism. Here, we present a protocol for designing and applying CATCH in mice. We describe how to integrate CATCH with *ex vivo* and *in vivo* electrophysiological recordings, as well as detail procedures for combining CATCH with behavioral assays. CATCH enables the specific control of neurotransmitter receptor function, offering a tool for both neurophysiology and behavioral studies.

For complete details on the use and execution of this protocol, please refer to Jehl et al.^1^

## Highlights

• CATCH enables precise, long-lasting inhibition of specific brain receptors
• Instructions for *ex vivo* electrophysiology using CATCH in brain slices
• Steps for *in vivo* acute electrophysiology coupled with CATCH
• Guide to applying CATCH *in vivo* for behavioral studies in mice

## Abstract (editor highlights)

Publisher’s note: Undertaking any experimental protocol requires adherence to local institutional guidelines for laboratory safety and ethics.

## Graphical abstract

**Figure:**
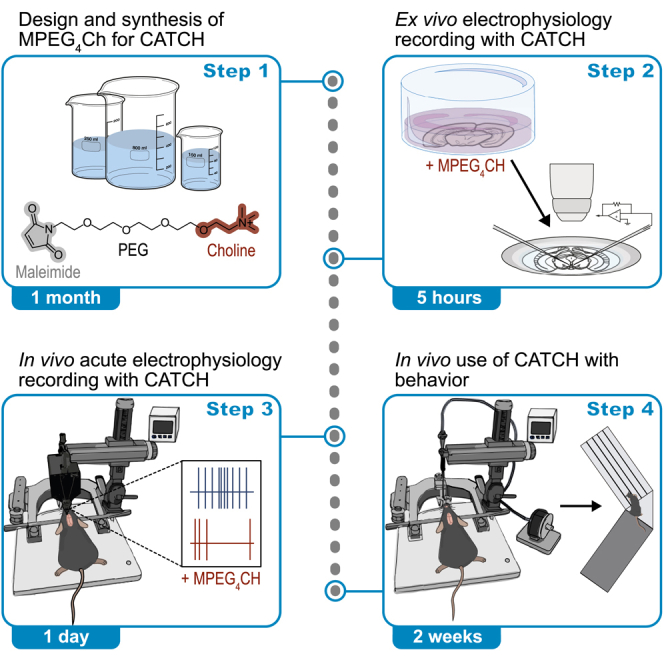

## Before you begin

Precise manipulation of endogenous receptors *in vivo* remains a significant challenge in neuroscience, particularly in the context of physiology and behavior, due to the lack of specific pharmacological tools and the difficulty in achieving sustained and localized effects. The application of traditional pharmacological tools is particularly problematic in *in vivo* electrophysiology, as compounds must be pressure-injected during experiments, often prematurely ending the recording.

The CATCH approach overcomes these limitations by enabling sustained, localized, and receptor-specific antagonism through a chemogenetic strategy, allowing for precise and long-lasting manipulation of neuronal signaling. This protocol is compatible with a variety of downstream applications, including *ex vivo* and *in vivo* electrophysiology and behavioral assays such as conditioned place preference, and can be adapted to other receptor systems with appropriate genetic targeting. For optimal reproducibility and interpretation, careful attention to experimental controls and validation steps is essential.

### Innovation

CATCH is a chemogenetic approach that enables sustained and receptor-specific antagonism *ex vivo* and *in vivo*. CATCH combines genetic receptor modification with a tethered ligand for irreversible binding. The genetic modification consists in introducing a cysteine residue that selectively reacts with maleimide-tethered ligands. CATCH is an extension of the photoswitchable tethered ligand (PTL) approach, in which light-sensitive ligand are covalently attached to cysteine-substituted ion channels or receptors.^2^^,^^3^^,^^4^^,^^5^^,^^6^^,^^7^^,^^8^^,^^9^^,^^10^^,^^11^^,^^12^^,^^13^^,^^14^^,^^15^^,^^16^ While PTLs offer reversible and rapid control of protein function, their *in vivo* use usually requires local light delivery within target brain regions. This can be technically demanding and often necessitates tethering animals to an optical fiber, which restricts movement. Moreover, many experimental settings do not require rapid on/off switching, and instead benefit from prolonged receptor antagonism. We demonstrated that CATCH achieves long-lasting (several days) and efficient (>80%) receptor blockade,^1^ making it particularly suitable for neurophysiological and behavioral studies. A key advantage of CATCH is the minimal genetic modification, which preserves native receptor pharmacology and allows investigation of receptor function within intact neural circuits. Although the cysteine substitution is not strictly bioorthogonal, no measurable off-target effects have been observed.^1^ This is provided that proper wild-type controls with the tethered ligand are included. This strategy has also been successfully applied to other systems, such as potassium channels, using cysteine-reactive quaternary ammonium groups^17^ and peptide toxins,^18^ further illustrating the versatility of this approach. Likewise, covalent activators could be deployed instead of antagonists, although this approach is limited to non-desensitizing receptors or ion channels.^19^

### Institutional permissions

All experiments on mice must first be approved by the appropriate institutional authority in accordance with the national guidelines. Our protocol was approved by the French Ministry of Education in Research and Paris Sciences et Lettres (PSL) University after consultation with the ethical committee #59 in accordance with the European Directive 2010/63/EU of the European Parliament.

### Design of the receptor-ligand pair

**Figure 1:**
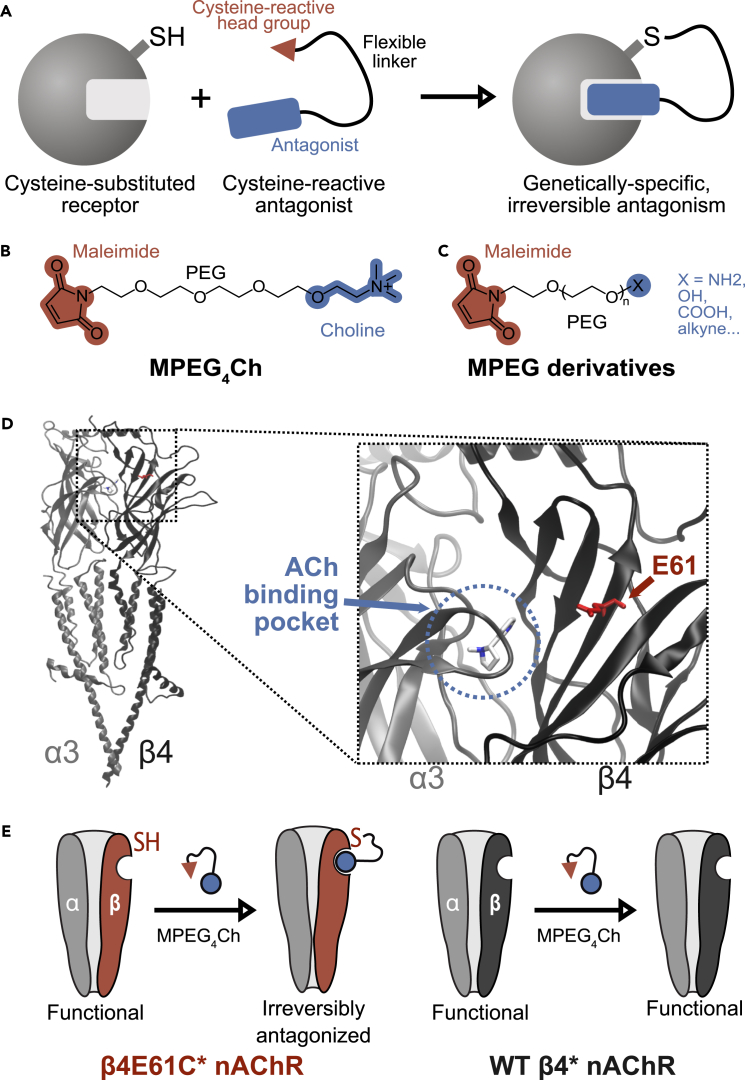
The CATCH method (A) In CATCH, a genetically-modified receptor incorporates a cysteine residue, for the covalent anchoring of a cysteine reactive antagonist. The tethered ligand is composed of a cysteine-reactive group, a flexible linker and a functional antagonist. After conjugation, the modified receptor becomes irreversibly antagonized. (B) Chemical structure of MPEG_4_Ch. (C) Chemical structures of MPEG derivatives with various PEG lengths (n) and headgroups, available from Broadpharm. Alkyne groups can be used for attaching more complex ligands through high-yield click chemistry.^20^ Example structures and additional information are available at: https://broadpharm.com/product-categories/Thiol-Reactive-Linkers/maleimide-linkers#mal-amido-peg-alkyne. (D) High resolution structure of the human α3β4 nAChR in complex with nicotine (PDB: 6pv7). Only a dimer is shown for clarity. Inset, zoom in on the ACh binding pocket, showing docked nicotine and amino acid E61 at close proximity, the position mutated to cysteine. (E) β4^E61C^-containing nAChRs are irreversibly antagonized after conjugation with MPEG_4_Ch, but not wild-type (WT) receptors.

CATCH is a chemogenetic method that relies on a uniquely selective receptor-ligand pair, in which a reactive antagonist is covalently tethered to a genetically-modified receptor bearing a strategically placed cysteine substitution. This engineered cysteine residue is positioned on receptor surface, in close proximity to the agonist binding site, to enable site-specific, irreversible antagonism upon covalent reaction with the ligand (Figure 1A).

1. Design of the ligand. The tethered antagonist is made up of three elements: a cysteine-reactive, maleimide head group; a flexible, water-soluble polyethylene glycol (PEG) linker; and a functional ligand (antagonist). Specifically, for manipulating nAChRs, we designed MPEG_4_Ch (maleimide - tetraethylene glycol – choline), which carries a four glycol unit PEG linker and a choline headgroup as an antagonist (Figure 1B).
  a. Reactive head group: maleimides remain the preferred cysteine-reactive group due to their excellent reactivity (they react within minutes with high yields),^21^ their biocompatibility and their commercial availability. Furthermore, maleimides-PEG linkers show excellent stability after bioconjugation,^22^ a key feature for irreversible antagonism.
  b. Linker: The length of the PEG linker is critical: a short linker (e.g., four units as in MPEG_4_Ch) ensures that the antagonist remains concentrated in the immediate vicinity of the binding pocket, thereby increasing local efficacy while minimizing off-target interactions. We recommend keeping the linker as short as possible.
  c. Pharmacophore: There is a tradeoff between using a minimal pharmacophore (the smallest chemical structure required for biological activity) and a high-affinity ligand. High affinity ligands improve binding specificity and may reduce side effects, but can be synthetically demanding. In contrast, minimal pharmacophores (e.g., the triethylammonium group in MPEG_4_Ch) benefit from facile synthesis, and their weak intrinsic binding is offset by covalent tethering, which ensures high local concentration.

***Note:*** Custom synthesis services are available from several companies. For example, Broadpharm offers a wide range of maleimide-PEG derivatives with various headgroups (Figure 1C)—including alcohols, amines, esters, amino acids, and alkynes (the latter enabling click chemistry for further modification). It is important to use compounds of high purity; in our case, Broadpharm specifies a purity of 98% for MPEG_4_Ch chloride powder.

2. Design of the cysteine-substituted receptor: On the receptor side, the cysteine mutation should be introduced at a surface-exposed position near the agonist binding site, but without altering receptor function in its unbound state. Identifying an optimal labeling site requires detailed knowledge of receptor structure and of the structure–activity relationships (SAR) of known ligands. For nAChRs, high-resolution structural models were previously used to design attachment points for photoswitchable ligands.^5^^,^^11^ The design of MPEG_4_Ch was guided by the structure of a previously developed photoswitchable antagonist, ensuring compatibility with existing cysteine-engineered nAChRs and expanding toolset versatility.^1^ We selected glutamate 61 (E61) in the β2 and β4 nAChR subunits, located at the extracellular entry of the acetylcholine binding pocket (Figure 1D). This mutation preserves native receptor function prior to ligand conjugation.^1^^,^^5^ Below we provide a general strategy for cysteine site selection.
  a. Identify candidate residues: Download a high-resolution structure from the Protein Data Bank (https://rcsb.org), ideally with a docked ligand. For nAChRs, the human α3β4 nAChR with nicotine (PDB: 6pv7)^23^ is suitable (Figure 1D). Because tethered ligands are flexible, complex docking is not required; instead, compute the distance between the docked ligand and candidate side chains using visualization software (e.g., VMD, https://www.ks.uiuc.edu/Research/vmd/).
  b. Filter candidates: Choose residues that are not essential for receptor function. Multi-sequence alignment (Clustal Omega https://www.ebi.ac.uk/jdispatcher/msa/clustalo?stype=protein) can help exclude highly conserved residues (marked ∗ or :) that are more likely to be functionally critical. Protein sequences in the FASTA format can be retrieved from the NIH database (https://www.ncbi.nlm.nih.gov/protein/).
  c. Introduce cysteine mutation: This can be performed with standard mutagenesis kits or outsourced to plasmid synthesis companies.
  d. Validate receptor function. Mutant receptors should be tested in functional assays (e.g., electrophysiology for nAChRs) to confirm that activity is preserved.
  e. Verify irreversible blockade. Once the covalent reaction is complete and excess reagent is washed out, the mutated receptors should be irreversibly blocked, with no further antagonist required to maintain the blockade, while WT receptors should remain unaffected (Figure 1E).

***Note:*** Comparison with other chemogenetics methods.

A direct variant of the CATCH method involves photoswitchable tethered ligands (PTLs), whose geometry can be modulated by light, enabling rapid and reversible control of protein function.^2^^,^^3^^,^^4^^,^^5^^,^^6^^,^^7^^,^^8^^,^^9^^,^^10^^,^^11^^,^^12^^,^^13^^,^^14^^,^^15^^,^^16^ These photoswitchable systems are particularly powerful for modulating synaptic transmission with high temporal precision. However, their application in behaving animals remains technically challenging due to the difficulty of delivering both the ligand and light locally. Moreover, they are not well suited for long-term experiments, as they often require mice to be tethered to external light sources, limiting behavioral flexibility. However, it should be noted that in the absence of illumination, some PTLs, the ones that bind to receptors in their dark-adapted state, act much like CATCH, producing sustained antagonism through covalent binding.^2^^,^^4^^,^^9^^,^^12^^,^^15^ However, this is not the case for light-controllable nAChRs, in which light is required to present the ligand in its binding pocket.^5^^,^^11^^,^^13^ Thus, while light delivery can present practical challenges in behaving animals, this does not diminish the fundamental advantage of PTLs in offering temporally precise, reversible control when optical stimulation is used.

The use of genetically introduced cysteine residues presents several advantages: it enables efficient and selective labeling with maleimides and generally results in minimal perturbation of receptor function. However, this strategy is not fully bioorthogonal, as endogenous cysteine residues are present on native proteins. As a result, non-specific binding of the ligand may occur. Although no off-target effects were observed in our study^1^ or with PTLs,^6^^,^^13^^,^^14^ it remains essential to perform control experiments using the tethered ligand in non-transfected or native tissue. To enhance labeling specificity, alternative tagging strategies have been developed.^24^ In particular, SNAP and CLIP tags offer high specificity for labeling genetically modified proteins.^25^^,^^26^^,^^27^ However, these tags are relatively large protein domains (∼20 kDa), and their fusion to membrane receptors may interfere with receptor folding or function. In the specific case of nAChRs and similar cys-loop receptors (i.e. GABA_A_, glycine and 5HT_3_ receptors), incorporation of tags at the N- or C-terminus alters receptor expression and activity,^28^ rendering this tagging approach inapplicable for this receptor class.

An entirely different chemogenetic strategy—the “bump–hole” approach—involves engineering a mutation (hole) in the receptor’s ligand-binding pocket, paired with a modified synthetic ligand (bump) that only fits the altered site.^29^ This approach has been successfully used to develop engineered GPCRs, including the widely known DREADDs (Designer Receptors Exclusively Activated by Designer Drugs),^30^^,^^31^ as well as cys-loop receptors^32^ that are activated selectively by synthetic ligands. While effective for remote and specific control of neuronal activity, these systems rely on receptors that no longer respond to their endogenous ligands, and are therefore unsuitable for studying the native physiological roles of these receptors in intact circuits.^24^

### Design of knockin mice or viral vectors

Two knock-in (KI) mouse lines, β2^E61C^ and β4^E61C^, were generated by targeted homologous recombination to introduce a single point mutation into the endogenous Chrnb2 or Chrnb4 gene. In these mice, all native β2 or β4 subunits are replaced by cysteine-substituted versions, ensuring unaltered receptor density, localization, and regulation under native promoters. This strategy preserves physiological expression patterns, but has a major limitation: the cysteine substitution is present in all cells expressing the targeted subunit, making it impossible to restrict covalent ligand labeling to specific cell types. Anatomical specificity can instead be achieved by local ligand infusion, though this does not provide cell-type resolution.

To achieve anatomical specificity, ligand delivery is performed locally, via stereotaxic infusion. However, a more refined approach would involve cell-type-specific replacement of endogenous nAChR subunits with cysteine-substituted variants. Although such a method is not yet widely established, recent advances in gene editing techniques offer a potential path forward.^33^

***Note:*** The KI mouse lines were generated on a C57BL/6N background by homologous recombination at the Phenomin core facility (Institut Clinique de la Souris, Illkirch, France). Exons 3 and 4 were fused to prevent alternative splicing at the E61 codon/splice acceptor site. Knockin mice were bred as homozygotes.

***Alternatives:*** Knockin mice can be produced by various academic or commercial facilities, such as GenOway (France), Polygene (Switzerland), Charles River (Global), or Cyagen (Global).

An alternative strategy is to deliver cysteine-mutated receptors via viral vectors. For instance, we used a lentivirus encoding pGK-β2^E61C^-IRES-eGFP to express β2^E61C^ receptors (available at Addgene, see key resources table for more information) to target expression of β2^E61C^ receptors in the ventral tegmental area.^13^ The single cysteine mutation E61C was inserted into pLenti-pGK-β2-IRES-eGFP by site-directed mutagenesis using the Quickchange II XL kit (Agilent). Mutations were verified by DNA sequencing.

This viral approach can be combined with MPEG_4_Ch, and offers crucial advantages such as restricting mutant expression to specific structures or cell types, thereby providing unparalleled manipulation of pre- versus postsynaptic receptors. We did not detect any change in overall nicotine-induced currents in transduced cells, suggesting no alteration in receptor number.^13^ This outcome likely reflects the fact that β2 subunits form obligate heteromers with α subunits, helping maintain total receptor numbers in balance. However, viral expression does not guarantee complete replacement of endogenous subunits, and stoichiometric alterations (two vs three β subunits per pentamer) could still affect receptor sensitivity to acetylcholine or nicotine. More generally, caution is warranted when applying this approach to homomeric receptors (e.g., α7 nAChRs), since ectopic expression may change total receptor number and complicate interpretation. In such cases, promoter strength becomes an important parameter to adjust expression levels and maintain physiological receptor density. Finally, viral strategies add technical requirements, including the need for an additional transduction step and thorough validation of receptor numbers.

***Note:*** When selecting and ordering viral vectors, always include a control virus. The control virus should express only the fluorophore and match the experimental virus in serotype, promoter, and recombinase dependence.

***Note:*** Upon receipt, thaw the virus and prepare aliquots. Store aliquots at −80°C for long-term stability, and use only one aliquot per experiment to avoid repeated freeze-thaw cycles. Thaw aliquots on ice before use and do not refreeze. AAV aliquots can be kept at 4°C for up to 6 months; lentivirus should be used immediately.

## Key resources table

**Table:** 

REAGENT or RESOURCE	SOURCE	IDENTIFIER
**Antibodies**		

Chicken polyclonal anti-GFP (1/1,000)	Sigma-Aldrich	Cat#AB16901, RRID:AB_90890
Alexa Fluor 488-conjugated anti-chicken IgY (H+L) (1/1,000)	Invitrogen	Cat#A-11039, RRID:AB_2534096

**Bacterial and virus strains**		

pIRES2-CMV-nAChRb2(E61C)-IRES-GFP	Durand-de Cuttoli et al.,^13^ Addgene	Cat#164779, RRID:Addgene_164779
pLenti-pGK-nAChRb2(E61C)-IRES-GFP	Durand-de Cuttoli et al.,^13^ Addgene	Cat#164777, RRID:Addgene_164777
AAV5-GFP: ssAAV5/2-hSyn1-EGFP-WPRE-hGHp(A)	ETH Zürich Viral Vector Facility	Cat#v81-5

**Chemicals, peptides, and recombinant proteins**		

MPEG_4_Ch chloride	Jehl et al.,^1^ custom-synthesized by Broadpharm	Cat#BP-24475, PubChem CID 146020404
MPEG_4_Ch stock solution	custom prep, see materials and equipment	N/A
Dimethyl sulfoxide (DMSO)	Fisher Scientific	Cat#D12345
Sodium chloride (NaCl)	Sigma-Aldrich	Cat#S7653
Nicotine hydrogen tartrate salt	Glentham LifeSciences	Cat# GL9693
Sodium hydroxide (NaOH)	Sigma-Aldrich	Cat#S5881
Potassium chloride (KCl)	Sigma-Aldrich	Cat#P9333
Monosodium phosphate (NaH_2_PO_4_)	Sigma-Aldrich	Cat#S8282
Calcium chloride (CaCl_2_)	Sigma-Aldrich	Cat#233506
Magnesium chloride (MgCl_2_)	Sigma-Aldrich	Cat#M2670
Sodium bicarbonate (NaHCO_3_)	Sigma-Aldrich	Cat#S6297
Glucose	Sigma-Aldrich	Cat#S0389
Sucrose	Sigma-Aldrich	Cat#49159
Kynurenic acid	Sigma-Aldrich	Cat#K3375
K-gluconate	Sigma-Aldrich	Cat#P1847
HEPES	Sigma-Aldrich	Cat#H3375
Ethylene glycol tetraacetic acid (EGTA)	Sigma-Aldrich	Cat#E3889
ATP magnesium salt (Mg-ATP)	Sigma-Aldrich	Cat#A9187
K-gluconate solution	custom prep, see materials and equipment	N/A
1x aCSF solution	custom prep, see materials and equipment	N/A
Sucrose solution	custom prep, see materials and equipment	N/A
Ketamine (Imalgene 1000)	Merial	N/A
Xylazine (Rompun 2%)	Bayer	N/A
Heparin	Sigma-Aldrich	Cat#PHR8927
Chloral hydrate	Sigma-Aldrich	Cat#302-17-0
Isoflurane	Piramal Healthcare	N/A
Vaseline	Vaseline	N/A
Mineral oil	Carlo Erba	Cat#356601
Vybrant Multicolor Cell-Labeling Kit (DiO, DiI, DiD solutions, 1 mL each)	Invitrogen	Cat#V22889
PBS 10X	Sigma-Aldrich	Cat#70011044
4% Paraformaldehyde (PFA)	Thermo Scientific	Cat#J19943.K2

**Experimental models: Organisms/strains**		

Mouse: C57BL/6NRj (males and females, 8–12 weeks)	Janvier Labs	RRID:IMSR_RJ:C57BL-6JRJ
Mouse: Chrnb4^E61C^ (males and females, 8–12 weeks)	Jehl et al.,^1^ provided by Phenomin	N/A

**Software and algorithms**		

Clampfit and Clampex (part of pClamp 10.6 suite)	Molecular Devices	RRID:SCR_011323
Cheetah software	Neuralynx	Cat#36-0301-0054, https://neuralynx.fh-co.com/research-software/cheetah/
CSC Spike Extractor software	Neuralynx	N/A
SpikeSort3D software	Neuralynx	https://neuralynx.fh-co.com/research-software/spikesort-3d/
R Statistical Software	https://www.r-project.org	RRID:SCR_001905
ezTrack	Pennington et al.^34^	RRID:SCR_021496
Fiji	Schindelin et al.^35^	RRID:SCR_002285
DMC-BrainMap	Jung et al.^36^	https://github.com/hejDMC/napari-dmc-brainmap

**Other**		

QuikChange II XL Site-Directed Mutagenesis Kit	Agilent	Cat#200522
Practum laboratory balance, model 224	Sartorius	Cat#practum224-1S
Spoon spatula	Dutscher	Cat#3340
PIPETMAN P10 micropipette	Gilson	Cat#F144802
PIPETMAN P1000 micropipette	Gilson	Cat#F144059M
Microcentrifuge tube, 1.5 mL	Eppendorf	Cat#0030108116
Microtube, 0.2 mL	Eppendorf	Cat#0030124332
Microtube, 0.5 mL	Eppendorf	Cat#0030123603
Laboratory bottle, 1 L	Schott Duran	Cat#218015455
Vortex Genie 2	Scientific Industries	Cat#SI-0256
25 mm sterile nylon membrane, 0.2 μm pore size	Nalgene	Cat#726-2520
Bottle-top sterile filter, 0.2 μm pore size	Nalgene	Cat#Z358223
Magnetic hotplate stirrer	LMS	Cat#hts-1003
Magnetic stir bar	Fisherbrand	Cat#001.3001
SevenEasy pH meter	Mettler Toledo	Cat#S20
Sonicator	BPAC	Cat#bpac03sk120s
Parafilm tape M	Amcor	Cat#9170002
Luer Lock syringe, 50 mL	Terumo	Cat#SS+50L1
Peristaltic pump Ismatec IPC-N-08	Ismatec	Cat#ISM936D
Compresstome VF-200	Precisionary Instruments	Cat#VF-200
Costar 12-well clear TC-treated multiple well plates	Corning	Cat#3513
P-87 Flaming/Brown micropipette puller	Sutter Instruments	Cat#P87
Thin-wall borosilicate glass	Warner Instruments	Cat#G150TF-3
Microloader	Eppendorf	Cat#EP5242956003
Axoclamp 200B Microelectrode amplifier	Molecular Devices	Cat#AxOPATCH 200B-2
Digidata 1550A Low-Noise Data Acquisition Digitizer	Molecular Devices	Cat#5035919A
Micromanipulator	Scientifica	Cat#PS-7550
Picopritzer Pneumatic pump	World Precision Instruments	Cat#PV800
GeneClamp 500 amplifier	Axon Instruments	N/A
8-way, 12-way, and 2-way 4-port electric rotary valve	Bio-Chem Fluidics	https://www.arcmedgroup.com/pumps-valves#electric-rotary-valves
SliceScope upright microscope	Scientifica	Cat#S-Scope-II
ROLERA Bolt Scientific CMOS Camera	QImaging	Cat#01-ROL-BOLT-M-12
Halogen lamp unit	Olympus	Cat#TH4-200
PIPETMAN P2 micropipette	Gilson	Cat#F144801
Gas anesthesia station for small animals	TEMSEGA	Cat#MiniHUB-V3
Charcoal anesthesia gas filter	F/AIR	Cat#80120
Dual Small Animal Stereotaxic w/ Digital Display	David Kopf Instruments	Cat#942-WA
ThermoStar Homeothermic System	RWD	Cat#69027
Routine stereo microscope	Leica Biosystems	Cat#M80
Drilling machine	Foredom	Cat# K.1070
0.7 mm steel drill burrs	Fine Science Tools	Cat#19009-07
Isis rodent shaver	Aesculap	Cat#GT421
100 μL, Gastight syringe, small removable needle	Hamilton	Cat#1710
Polyethylene Tubing	Instech	Cat#BTPE-10
Loctite Superglue-3 original	Henkel	Cat#2959596
ToughCut fine scissors	Fine Science Tools	Cat#14058-09
Dumont #5 forceps	Fine Science Tools	Cat#11251-20
Olsen-Hegar needle holders with suture cutters	Fine Science Tools	Cat#12002-12
Instrument sterilization container	Fine Science Tools	Cat#20840-02
Autoclave	Matachana	Cat#S1000
Thomas Tetrode Mini Matrix microdrive system	Thomas Recording	Cat#AN000423
Tetrode for Tetrode Mini Matrix (Tip A)	Thomas Recording	Cat#AN000259
Quartzglass microinjection pipettes for Thomas microdrive	Thomas Recording	Cat#AN000155
Digital Lynx 4SX	Neuralynx	Cat#31-0605-0141, https://neuralynx.fh-co.com/research-hardware/data-acquisition/digital-lynx-sx/
PC-10 puller	Narishige	RRID:SCR_022057
Capillary micropipette for PCR 10 μL	Drummond	Cat#5-000-1001-X10
One-axis Oil Hydraulic Micromanipulator	Narishige	Cat#MO-10
Y-maze apparatus	Imetronic	https://www.imetronic.com/devices/y-maze/
Intel RealSense Camera	Intel	Cat#D455
Surfa Safe Premium disinfectant spray	Laboratoires Anios	Cat#2419326
Vibratome VT1000 S	Leica Biosystems	RRID:SCR_016495

## Materials and equipment

### Preparation of MPEG_4_Ch stock solution

• To prepare 0.5 mL of a 50 mM stock solution of MPEG_4_Ch retrieve MPEG_4_Ch chloride (MW = 394.9 g/mol) powder stored at −20°C.

***Note:*** As a general rule, chemicals should be stored at the highest practical concentration (here 50 mM). However, a lower concentration (e.g. 10 mM) is also acceptable.

***Note:*** Salts—such as the chloride salt added to MPEG_4_Ch in this case—are generally more soluble in water than their uncharged counterparts. This increased solubility makes them preferable for *in vivo* use.

• Weigh 9.87 mg of MPEG_4_Ch chloride using a clean, dry spatula.
• Transfer the powder into a sterile microcentrifuge tube.
• Just before use, open a fresh ampoule of anhydrous dimethyl sulfoxide (DMSO).

**CRITICAL:** DMSO must be strictly anhydrous. Maleimide groups in MPEG_4_Ch react with water, compromising compound integrity. Do not use previously opened DMSO, even if tightly capped, as it may have absorbed moisture from the air.

• Add 500 μL of the freshly opened anhydrous DMSO to the tube containing MPEG_4_Ch.
• Mix gently by pipetting up and down or vortexing until fully dissolved.
• Aliquot 10 μL portions into clearly labeled 0.2 mL Eppendorf tubes.

***Note:*** Store aliquots at −20°C and minimize air exposure by using one aliquot per experiment and keeping tubes sealed. Avoid repeated freeze-thaw cycles.

### Preparation of filtered 0.9% saline solution

• Add 9 g of Sodium Chloride (NaCl; Sigma-Aldrich S7653) and 950 mL of milli-Q water to a 1 L bottle.
• Stir at medium speed until fully dissolved and adjust the volume up to 1 L with milli-Q water.
• Filter the solution using a 50 mL syringe (Terumo Luer Lock Syringe, 50 mL) and an appropriate syringe filter, such as Nalgene 25 mm syringe filter (Sterile Nylon membrane, 0.2 μm pore size).

***Note:*** Store the bottle at 4°C (up to 1 year – date the bottle).

### Preparation of nicotine tartrate solution (57 mg/L)

• Pour 100 mL of sterile, filtered 0.9% saline into a clean beaker.
• Weigh and add 5.7 mg of nicotine hydrogen tartrate salt (Glentham LifeSciences, GL9693, MW = 462.41 g/mol) to the saline, yielding a final concentration of 57 mg/L.
• Stir the solution thoroughly until the salt is completely dissolved.
• Adjust the pH to 7.2 ± 0.2 using sodium hydroxide (NaOH) as needed.

***Note:*** This solution is designed to deliver a dose of 0.2 mg/kg nicotine free base per injection, using an injection volume of 0.1 mL per 10 g body weight (for a 25 g mouse, inject 0.25 mL). This corresponds to an injection volume of 10 mL/kg.

***Note:*** Prepare the 57 mg/L solution fresh during the week of the experiment, or alternatively, prepare a more concentrated stock solution, aliquot, and store at −20°C. Thaw individual aliquots and dilute to the working concentration as needed.

### Preparation of artificial cerebrospinal fluid (aCSF) 10x stock solution

**Table:** 

Reagent	Final concentration (mM)	Amount
milli-Q water	–	1 L
NaCl	1250	73.05 g
KCl	25	1.86 g
NaH_2_PO_4_	12.5	1.5 g
NaHCO_3_	260	21.85 g

***Note:*** Place a clean 1 L beaker with a magnetic stir bar on a stirring plate and add 500 mL of milli-Q water. While stirring, add NaCl, KCl, NaH_2_PO_4_, and NaHCO_3_. Continue stirring until all components are fully dissolved. Once everything is dissolved, transfer the solution into a clean 1 L volumetric flask and fill up to the 1 L mark with milli-Q water. Place a 0.2 μm membrane bottle-top filter (Nalgene) on a clean 1 L collection bottle, connect it to a vacuum source, and pour the solution into the filter reservoir. Allow the solution to fully filter into the bottle. Label the final solution appropriately and store as needed. Store the final solution at 4°C and use within 30 days.

### Preparation of aCSF 1x

**Table:** 

Reagent	Final concentration (mM)	Amount
milli-Q water	N/A	1 L
CaCl_2_ 2H_2_O 2 M	2	1 mL
MgCl_2_ 6H_2_O 2 M	1	0.5 mL
Glucose	10	1.98 g
Sucrose	15	5.13 g
aCSF 10x stock solution	N/A	100 mL

***Note:*** Place a clean 1 L beaker on a magnetic stirring plate and add a clean magnetic stir bar. Pour 500 mL of milli-Q water into the beaker. While stirring, add the CaCl_2_ and MgCl_2_ stock solutions, then weigh and add glucose and sucrose. Stir until fully dissolved. Using a volumetric flask, measure and add the aCSF 10x stock solution to the beaker. Rinse the volumetric flask with a small amount of milli-Q water and pour the rinse into the beaker to recover any remaining aCSF 10x stock solution. Transfer the contents of the beaker into a clean 1 L volumetric flask and fill up to the 1 L mark with milli-Q water. Mix by gently inverting the volumetric flask several times, then transfer the final solution into a clean 1 L bottle for storage. Store the final solution at 4°C and use within 3 days.

### Preparation of sucrose-based cutting solution

**Table:** 

Reagent	Final concentration (mM)	Amount
milli-Q water	N/A	1 L
MgCl_2_ 6H_2_O 2 M	5.9	2.95 mL
Glucose	2.5	0.49 g
Sucrose	25	8.55 g
aCSF 10x stock solution	N/A	100 mL
Kynurenic Acid	N/A	200 mg

***Note:*** Place a clean 1 L beaker on a magnetic stirring plate and add a clean magnetic stir bar. Pour 500 mL of milli-Q water into the beaker. While stirring, add the MgCl_2_ stock solution. Weigh and add the glucose and sucrose. Using a 100 mL volumetric flask, measure and add 100 mL of aCSF 10x stock solution to the beaker. Rinse the volumetric flask with a small amount of milli-Q water and pour the rinse into the beaker to recover any remaining 10x aCSF. Transfer the contents of the beaker into a clean 1 L volumetric flask and fill to the 1 L mark with milli-Q water. Mix by gently inverting the volumetric flask several times, then transfer the solution into a clean 1 L storage bottle. Weigh 200 mg of kynurenic acid and add it to the solution. Sonicate for 15 min to ensure complete dissolution. Store the final solution at 4°C and use within 3 days.

### Preparation of K-gluconate internal solution

**Table:** 

Reagent	Final concentration (mM)	Amount
milli-Q water	N/A	50 mL
K-gluconate	135	1.5812 g
HEPES (free acid)	10	119.15 mg
KCl	5	18.6375 mg
MgCl_2_	2	20.33 mg
EGTA	0.1	1.9018 mg
Mg-ATP	2	50.718 mg

***Note:*** Place a clean 50 mL glass beaker on a magnetic stir plate and add a magnetic stir bar. Pour 35 mL of milli*-Q* water into the beaker and begin stirring. While stirring, weigh and add the following components in sequence: K-gluconate, HEPES (free acid), KCl, MgCl_2_, EGTA, and Mg-ATP. Stir until all solids are fully dissolved. Adjust the pH to 7.2 using small volumes of HCl (e.g., 0.1–1 M), monitoring with a calibrated pH meter. Transfer the solution to a clean 50 mL volumetric flask and bring the final volume up to 50 mL with milli-Q water. Seal the flask with Parafilm and mix gently by inverting several times. The solution should then be filter-sterilized using a 0.2 μm pore syringe filter (Nalgene) into a sterile container. Aliquot 0.5 mL of the internal solution into sterile, labeled 0.5 mL Eppendorf tubes. Store aliquots at −20°C and use within 6 months. Use thawed aliquots on the day of the experiment. Do not refreeze. Discard any remaining solution after the recording session to avoid degradation or contamination.

### Preparation of choral hydrate solution for anesthesia

• To prepare 100 mL of an 8% (w/v) solution, weigh out 8 grams of chloral hydrate powder (Sigma-Aldrich 15307).
• Add the powder to 80 mL of milli-Q water in a beaker or flask.
• Stir the solution using a magnetic stir bar until the powder is completely dissolved.
• Transfer the solution to a graduated cylinder or volumetric flask and add milli-Q water to bring the final volume up to 100 mL.
• Filter the solution through a 0.22 μm sterile syringe filter into a sterile container. Store at 4°C and protect from light.

***Note:*** Chloral hydrate is hazardous and should be handled with appropriate PPE. Safer alternatives, such as isoflurane, may be used if preferred or required by institutional guidelines.

### Surgical setup for *in vivo* electrophysiology and MPEG_4_Ch injection

**Timing: 30 min**

• Surgical Instruments and supplies.
  ○ Pulled glass micropipettes (Drummond 10 μL PCR Pipettes): For microinjections.
  ○ Microinjector (e.g., Narishige MO-10 or IMS-30).
  ○ P2 micropipette: For precise liquid handling.
  ○ Parafilm: For preparing and handling small volumes of solution to infuse.
  ○ Mineral oil: For filling micropipettes and preventing evaporation.
  ○ Vaseline: For lubricating pipette plungers.
  ○ Shaver: For removing fur from the surgical area.
  ○ Heating pad/mat (set to 37°C): To maintain animal body temperature during surgery.
  ○ Stereotaxic frame (e.g., Kopf) with stereotaxic arm, ear bars and gas mask: For head fixation and anesthesia delivery.
  ○ Stereo microscope (e.g. Leica M80): for precise visualization.
  ○ Fine scissors and forceps (e.g., FST instruments): For precise tissue handling and dissection.
  ○ Drilling machine with 0.9 mm steel drill bit: For craniotomy.
  ○ Sutures thread with needle and needle holders: For wound closure.
  ○ Cotton buds: For cleaning and hemostasis.
  ○ Betadine: For skin disinfection.
  ○ Physiologic gel: To protect eyes from drying during anesthesia.
  ○ Sterile gloves: For aseptic technique.
• Anesthesia and Fluids.
  ○ Isoflurane: For inhalation anesthesia (3%–4% for induction and 1%–2% for maintenance).
  ○ Oxygen source: For delivering anesthetic gases.
  ○ Ketamine (100 mg/mL): Used for injectable anesthesia, typically combined with xylazine.
  ○ Xylazine (Rompun, 20 mg/mL): Administered in combination with ketamine for deep surgical anesthesia.
  ○ 0.9% Saline: For hydration and solution preparation.
  ○ Lidocaine: For local anesthesia at incision site. Prepare a solution at 1 mg/mL (by diluting 10 μL of 10 mg/mL stock in 90 μL saline). Inject 0.125 mL per animal, corresponding to a dose of 5 mg/kg (for a 25 g mouse, this is 0.005 mL/g).
  ○ Buprenorphine: For perioperative analgesia. Prepare a solution at 0.03 mg/mL (by diluting 20 μL of stock solution in 180 μL of physiological saline). Inject 0.05 mL (50 μL) subcutaneously per mouse, corresponding to a dose of 0.06 mg/kg (for a 25 g mouse, this is 0.002 mL/g).
  ○ MPEG_4_Ch stock solution (700 nl, 50 mM in water-free DMSO, −20°C): For CATCH application.
  ○ AAV5-GFP: For injection site verification via fluorescence. AAV5-GFP refers to the ssAAV5/2-hSyn1-EGFP-WPRE-hGHp(A) viral vector (see key resources table). The viral stock used had a titer of 5.7 × 10^11^ vg/mL.
  ○ Fluorescent dyes (DiI, DiO, DiD; 1 mg/mL): For visualizing injection sites.
• Sterilization and Storage.
  ○ Autoclave: For sterilizing surgical instruments.
  ○ Sterile storage containers: For keeping tools and reagents contamination-free.
  ○ Charcoal anesthesia gas filter: For isoflurane waste gas scavenging.

***Note:*** Prepare all instruments and reagents before beginning surgery to minimize anesthesia time. Sterilize surgical instruments by autoclaving them beforehand at 121°C for 30 min. Maintain aseptic technique throughout all surgical procedures. Ensure all solutions are freshly prepared and filtered as described in the protocol.

## Step-by-step method details

### *Ex vivo* electrophysiology recording after incubation with MPEG_4_Ch

**Timing: 5 h**

**Timing: 20–30 min for step 1**

**Timing: 20 min for step 2**

**Timing: 15 min for step 3**

**Timing: 3–4 h for step 4**

This procedure describes how to assess the efficacy and specificity of CATCH-mediated receptor antagonism at the cellular level using acute brain slices. As an example, this approach was employed in Jehl et al.^1^ to quantify the impact of MPEG_4_Ch incubation on nAChR function in the interpeduncular nucleus (IPN) of Chrnb4^E61C^ KI mice.

***Note:*** By comparing nicotine-evoked currents in slices treated with MPEG_4_Ch versus untreated controls, this method provides a direct and quantitative readout of receptor blockade and functional receptor expression levels.

**CRITICAL:** This *ex vivo* validation is critical for confirming the molecular and functional specificity of CATCH prior to *in vivo* or behavioral applications.

**Figure 2:**
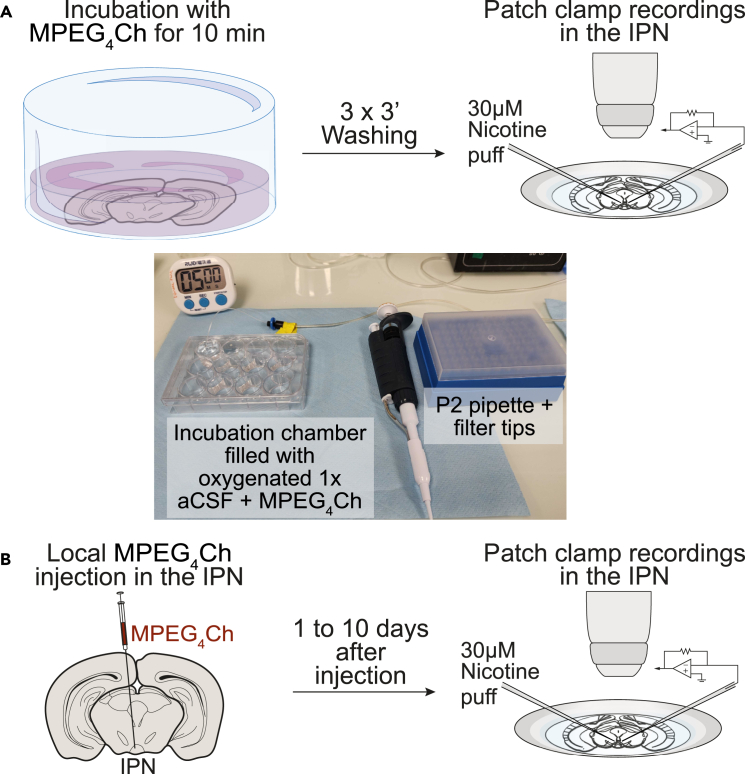
*Ex vivo* electrophysiological recordings with CATCH (A) Acute brain slices are incubated with 50 μM of MPEG_4_Ch for 10 minutes under oxygenation, then rinsed three times with fresh 1X aCSF before immediate transfer to the patch clamp chamber. (B) Alternatively, MPEG_4_Ch can be injected *in vivo* in KI mice, allowing direct patch-clamp recordings from acute slices without further incubation.
3. Patch-clamp electrophysiology: Pre-recording preparation.
  a. Pipette preparation: Pull patch pipettes (3–6 MΩ) from borosilicate glass capillaries (G150TF-3, Warner Instruments) using a micropipette puller (P-87, Sutter Instruments, Novato, CA).
    ***Note:*** This resistance range is suitable for whole-cell recordings in most neurons, though slight adjustments may be required based on cell type and morphology.
  b. Thaw an aliquot of K-gluconate internal solution (preparation detailed in materials and equipment section).
    i. To fill pipettes with intracellular solution, draw the aliquot into a 1 mL syringe fitted with a 4 μm Nalgene syringe filter and a microloader (Eppendorf).
    ii. Fill pipettes until the solution reaches the electrode tip, then gently flick the pipette to remove any air bubbles.
      ***Note:*** Use the same method to fill puff pipettes with nicotine-containing 1x aCSF (30 μM), but omit the syringe filter.
  c. Perfusion and positioning:
    i. Power on the patch clamp rig (Axopatch 200B amplifier connected to a Digidata 1550 Low-Noise acquisition system, Molecular Devices, USA) and acquisition software (Clampex software, pClamp 10.5, Molecular Devices).
    ii. Begin perfusion of fresh oxygenated 1x aCSF into the recording chamber at a flow rate of 0.5–1 mL/min via gravity feed or peristaltic pump.
    iii. Direct the waste line into a 2 L Erlenmeyer flask containing diluted bleach, connected to a vacuum line.
  d. Once perfusion is stable, use a transfer pipette to gently place the slice in the recording chamber.
  e. Secure the slice with a stainless-steel slice anchor (harp) fitted with nylon threads.
  f. Mount the patch and puff pipettes onto their respective headstages.
  g. Check slice viability, troubleshooting 1.
4. Patch-clamp electrophysiology: Patching.
  a. Apply positive pressure to the patch pipette using a syringe.
  b. Lower both pipettes into the recording bath.
  c. Confirm the resistance of the recording pipette is 3–6 MΩ.
  d. Bring both pipettes above the selected neuron using micromanipulators (Scientifica) and zero the pipette offset using the “membrane test” function on the Clampex software.
  e. Slowly descend the patch pipette until a small dimple and increased resistance are observed.
  f. Release the positive pressure and look for a further resistance increase.
  g. Gently apply suction via the syringe while holding the potential at −40 mV to form a giga-ohm seal.
  h. Once the seal is stable, hold the membrane potential at −70 mV and apply brief, gentle suction to break into whole-cell configuration.
    ***Note:*** Monitor the capacitive transient and adjust compensation on the amplifier as needed when breaking into whole-cell mode.
  i. Set the amplifier to **voltage-clamp mode** and begin recording using a **gap-free** protocol.
  j. Position the puff pipette ∼20 μm from the patched cell and adjust its height to match the patch pipette.
  k. Use a picopritzer pneumatic pump (PV800, WPI) connected to a pressurized air system to deliver puffs of nicotine (30 μM) at a starting pressure of 5–10 psi.
    ***Note:*** Start with minimal puff pressure and adjust carefully to avoid disturbing the patched cell. The first puff typically delivers a lower concentration of nicotine due to aCSF diffusion into the pipette; therefore, 2–3 puffs may be needed to ensure stable and reliable responses. Troubleshooting 2.
  l. Following recordings, clean all tools, tubing, and perfusion lines with milli-Q water and, where appropriate, 70% ethanol.
5. Data export and analysis.
  a. Export raw current traces from Clampfit or compatible acquisition software.
  b. Analyze peak nicotine-induced current amplitudes in R or Python, depending on the experimental goal. Troubleshooting 3.
  c. Example Goal: Compare peak current amplitudes in IPN neurons between MPEG_4_Ch-treated and untreated (control) slices in KI mice.

***Note:*** The statistical method and sample size depend on the specific hypothesis. For comparisons, use *t* tests or linear mixed-effects models as appropriate.

1. Dissection and slice preparation.^37^
  a. Prepare the dissection setup.
    **CRITICAL:** Ensure all solutions (sucrose-based cutting solution, 1x aCSF) are freshly prepared in the morning, stored appropriately (max 3 days at 4°C), and fully saturated with carbogen gas (95% O_2_/5% CO_2_).
  b. Chill the cutting solution to ≈0°C in the freezer.
    ***Note:*** Keep the cutting solution on ice throughout the dissection and slicing steps**.** The detailed procedure for preparing these solutions can be found in the materials and equipment section of this protocol.
  c. Anesthetize a KI mouse via intraperitoneal injection of ketamine/xylazine (0.11 mL ketamine [100 mg/mL] + 0.06 mL xylazine [20 mg/mL] in 0.63 mL sterile NaCl). Confirm anesthesia via loss of pedal reflex before proceeding.
  d. Perform transcardiac perfusion:
    i. Open the thoracic cavity.
    ii. Inject 0.1 mL of heparin (1000 U/mL) into the apex of the heart.
    iii. Clamp the descending aorta.
    iv. Perfuse with ice-cold, oxygenated sucrose-based cutting solution using a peristaltic pump (Ismatec IPC) for 1 min 20 sec.
  e. Decapitate the mouse and extract the brain.
  f. Immediately submerge the brain in 0°C oxygenated cutting solution.
  g. Place the brain ventral side down on filter paper and make two coronal cuts: one behind the olfactory bulbs and one through the cerebellum.
    ***Note:*** This protocol targets the IPN; adjust cutting if targeting other regions. IPN coordinates can range between Antero-Posterior (AP) −3.3 to −3.6 mm; Medio-lateral (ML) 0.35–0.5 mm; Dorso-ventral (DV) 4.2–4.9 mm, from Bregma.
  h. Affix the rostral brain section onto the specimen holder of a Compresstome using glue and supportive agarose gel.
  i. Fill the slicing chamber with 150 mL ice-cold oxygenated cutting solution.
  j. Slice the brain into 250 μm coronal sections using the Compresstome.
  k. Transfer slices containing the IPN into a 35°C oxygenated sucrose-based recovery solution for 8 minutes. Repeat for additional IPN slices (typically 2–3 slices per brain).
  l. Move slices to oxygenated 1x aCSF (kept at 20°C–25°C).
    i. Incubate for at least 60 minutes.
  m. Clean all dissection instruments thoroughly with distilled water and, when appropriate, 70% ethanol.
2. MPEG_4_Ch incubation (Figure 2A).
  ***Note:*** This protocol is optimized for between-subject comparisons. Ideally, control and MPEG4Ch-treated slices should be recorded on the same day, with control recordings performed first, because a slice cannot serve as its own control after MPEG4Ch incubation. However, in practice, we found that slice health can decline over the course of a recording session, potentially confounding the results. To minimize this issue, we performed control and treated recordings on separate days, ensuring that each slice was in optimal condition at the time of recording.
  ***Note:*** As an alternative to in-slice MPEG_4_Ch incubation, MPEG_4_Ch can be locally injected in the IPN *in vivo* in KI mice (Figure 2B). Patch-clamp recordings can then be performed on acute brain slices without further incubation, since the compound is already present in the tissue (see step 17). Our *ex viv*o data show that MPEG_4_Ch-mediated antagonism of β4 nAChR subunits is detectable as early as 1 day after infusion and lasts for at least 10 days^1^; longer durations should be validated experimentally.
  a. Freshly prepare 700 μL of MPEG_4_Ch-containing 1x aCSF by diluting 0.7 μL of 50 mM MPEG_4_Ch stock (in DMSO) to achieve 50 μM final concentration.
    **CRITICAL:** Prepare the 50 μM MPEG_4_Ch solution fresh on the day of the experiment, since maleimide groups in MPEG_4_Ch react with water, compromising compound integrity. Store the MPEG_4_Ch stock solution (50 mM in DMSO) at −20°C. Avoid repeated freeze-thaw cycles by aliquoting the stock solution as needed.
    ***Note:*** Lower concentrations, such as 10 μM, should also be effective, but concentrations higher than 50 μM are not recommended for slice experiments.
  b. After the recovery period, transfer the slice containing the IPN to a small incubation chamber (using a 12 well plates) filled with oxygenated 1x aCSF, continuously bubbled with 95% O_2_/5% CO_2_.
  c. Remove the existing 1x aCSF with a transfer pipette and immediately replace it with the 700 μL of MPEG_4_Ch -containing 1x aCSF, ensuring continuous oxygenation.
  d. Incubate the slice for 10 minutes under constant oxygenation.
  e. Rinsing: Remove the MPEG_4_Ch-containing solution and replace it with 700 μL of fresh oxygenated 1x aCSF. Allow the slice to rest for 3 minutes.
    i. Repeat this rinsing step two more times for a total of three rinses.
  f. Transfer the slice to the patch clamp recording chamber.
    ***Note:*** Handle the slice with extreme care to prevent damage during transfers and throughout the oxygenation process.

### *In vivo* acute electrophysiology recording after local infusion of MPEG_4_Ch

**Timing: 1 day**

**Timing: 5 min for step 7**

**Timing: 35 min for step 8**

**Timing: 5 min for step 9**

**Timing: 15 min for step 10**

**Timing: 5–10 min for step 11**

**Timing: typically, 3–6 h for step 12**

**Timing: 30 min for step 12b**

**Timing: typically, 1–2 h per dataset, depending on channel count and software for step 15**

This section describes how to apply the CATCH protocol to investigate the functional consequences of localized receptor antagonism on neuronal spiking activity. It describes the recording and analysis of neuronal activity in the IPN of anesthetized mice following local infusion of MPEG_4_Ch and subsequent nicotine injections, using tetrode-based electrophysiology.

***Note:*** This protocol can be adapted for use in other brain regions and with different receptor types.

6. Prior to beginning the procedure gather all the supplies listed in the materials and equipment section.
7. Prepare a fresh dilution of MPEG_4_Ch solution for *in vivo* use.
  a. Take MPEG_4_Ch stock solution (50 mM) stored at −20°C in water-free DMSO.
  b. Prepare 0.9% Saline Solution as described in materials and equipment section.
  c. Dilute MPEG_4_Ch Stock Solution.
    i. In a 1 mL Eppendorf tube, add 100 μL of filtered saline and 0.5 μL of MPEG_4_Ch stock solution (50 mM) to achieve a final concentration of 250 μM for *in vivo* use.

**CRITICAL:** Prepared MPEG_4_Ch solution should be used within the same day. Prepare a fresh dilution daily before use.

***Note:*** Ensure solution is at the desired concentration and well diluted (use vortex shakers).

**Figure 3:**
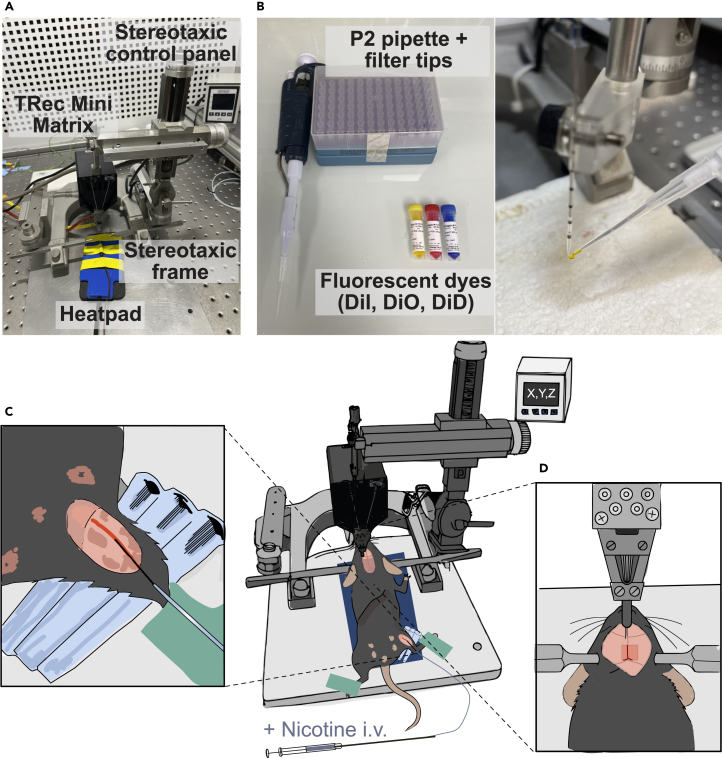
*In vivo* acute electrophysiological recordings with CATCH (A) Stereotaxic frame equipped with a Thomas Recording (TRec) Matrix, enabling precise injection and recording with multiple independently movable tetrodes. (B) Application of fluorescent dyes to the tip of a glass micropipette (or injection cannula) using a P2 pipette. This allows for post-hoc verification of the glass micropipette track to confirm accurate targeting of the brain region. (C) Catheterization of the saphenous vein for i.v. drug administration, such as nicotine. A needle tip attached to a plastic tube is inserted into the vein close to an 90° angle to avoid perforation. Adhesive tape on the paw and pipette tips placed behind the hind limb help to tense the leg, facilitating easier access to the saphenous vein. (D) Lowering the tetrodes into the brain following craniotomy and removal of the dura mater.

8. Anesthesia.
  a. Induce deep anesthesia with an intraperitoneal (i.p.) injection of chloral hydrate (8%, 400 mg/kg; 100 μL per 10 g body weight).
  b. Leave mouse in his transport cage for 30 min.
  c. When mouse is deeply anesthetized, secure the mouse in a stereotaxic apparatus (Figure 3A).

***Alternatives:*** Use isoflurane. If you choose this option, ensure that the anesthesia mask is connected to an anesthesia station (MiniHUB V3, TEMSEGA, France) with two separate tubes: one for the supply gas and one for the exhaust.^38^

***Note:*** Supplement anesthesia as needed with a 1/3x dilution to maintain stable anesthesia throughout the experiment.

9. Craniotomy.
  a. Perform a midline scalp incision with ToughCut fine scissors.
  b. Clean the area with cotton buds and sterile saline.
  c. Check the distance between bregma and lambda to ensure alignment of the skull in the anterior-posterior plane; measure left-right skull roll.
  d. Drill with a **0.9 mm steel drill bit** a craniotomy above the target site(s).

***Note:*** In our case we targeted the IPN: AP –3.3 to −3.6 mm; ML 0.35–0.5 mm; DV 4.2–4.9 mm. Use a 5° angle for all insertions into the IPN to avoid the superior sagittal sinus.

***Note:*** The craniotomy area should be sufficiently large to allow sampling from multiple coordinates within the target brain region during acute electrophysiology recordings. However, creating an excessively large window increases the risk of brain swelling or bleeding, which can compromise both the health of the animal and the quality of the recordings. Therefore, it is important to balance accessibility with minimizing tissue damage.

10. Cannula Insertion for local infusion of MPEG_4_Ch.
  a. Mount the Mini-Matrix with independently movable tetrodes and a stainless-steel injection cannula (OD 120 μm) onto the stereotaxic frame.
  b. Insert the injection cannula into the brain at the target coordinates and wait 5 min for stabilization of the brain tissue.
    ***Note:*** Check the mobility of tetrodes and cannula prior to surgery; replace if necessary.
    ***Alternatives:*** For local infusion, you can use a glass micropipette connected to a microinjector or a One-axis Oil Hydraulic Micromanipulator.
  c. To verify flow, gently expel a small drop of liquid from the cannula tip. If the cannula appears clogged, moisten the tip with filtered saline and try again. If the issue persists, replace the cannula.
  d. Infuse MPEG_4_Ch (250 μM; 700 nL for IPN) at a rate of 10 nL/s using the microinjector attached to the cannula.
  e. Wait 5 min after infusion before retracting.
  f. Verify cannula flow after the injection to confirm successful delivery.
    ***Note:*** For control experiments, infuse an equivalent volume of filtered saline.
    ***Note:*** For maleimide-thiol reactions performed *in vitro*, concentrations in the tens of micromolar range are typically sufficient. However, for *in vivo* applications, higher concentrations—usually in the hundreds of micromolar—are used to compensate for immediate dilution upon administration.
    **CRITICAL:** Always confirm injection cannula placement post hoc with histology. To check placement, apply a fluorescent dye (DiI, DiO, or DiD at the stock concentration of 1 mg/mL) to the cannula tip about 5 min before entering the brain (Figure 3B).
  g. Wait 2 hours post-infusion before starting electrophysiological recordings.
11. Venous Catheterization (Figure 3C).
  ***Note:*** Venous catheterization is performed to enable precise, repeatable intravenous (i.v.) administration of nicotine (or saline) during *in vivo* electrophysiological recordings. This approach allows for rapid and controlled delivery of nicotine directly into the bloodstream, ensuring consistent pharmacokinetics and minimizing stress or movement artifacts that could occur with repeated manual injections.
  a. Preparation. Prepare all materials before beginning this step to minimize the duration of anesthesia and reduce risk of complications.
    i. Fill a 1 mL syringe connected to a 30G needle with sterile saline.
    ii. Connect the syringe to a length of PE10 tubing.
      ***Note:*** The length of the PE10 tubing determines the volume delivered. For example, 16.2 cm of PE10 tubing (internal radius 0.014 cm) corresponds to 10 μL, calculated as:
      $$Length=\frac{Volume}{\left(\pi \times {radius}^{2}\right)}$$
    iii. Detach the thin metal shaft of a 30G needle from its plastic base; this metal shaft will be inserted into the vein.
    iv. Insert the metal shaft of the 30G needle in the other end of the PE10 tubing.
    v. Prepare a separate 100 μL Hamilton syringe for subsequent intravenous administration of nicotine or saline.
  b. Surgical Exposure.
    i. Using ToughCut fine scissors, make a small incision in the skin on the inner side of the hind leg to expose the underlying tissue.
    ii. Carefully separate and remove the thin connective tissue layer between the skin and muscle with two Dumont #5 forceps to fully expose the saphenous vein.
      ***Note:*** Use fine forceps and scissors for precise dissection to avoid damaging the vein.
  c. Catheter Insertion.
    i. Gently insert the 30G needle shaft (connected to PE10 tubing) into the saphenous vein.
    ii. Slowly inject a small volume of saline to confirm that the solution flows freely into the vein (no resistance or swelling at the site). Troubleshooting 4.
  d. Once correct placement is confirmed, apply a small amount of SuperGlue at the entry point to secure the needle and prevent movement during the experiment.
    **Caution:** Avoid excess glue, which may occlude the vein or adhere to surrounding tissue.
  e. Disconnect the saline syringe and connect the PE10 tubing to the prepared 100 μL Hamilton syringe containing nicotine or saline for i.v. administration during the experiment. Troubleshooting 5.
12. Electrophysiological Recordings.
  a. Before inserting the tetrodes, carefully remove the dura mater overlying the target region using a 90°-bent needle or fine forceps.
    **Tip:** Gently lift and cut the dura to avoid damaging underlying brain tissue. Keep the area moist with sterile saline throughout. In case of bleeding, control it with gentle pressure with hemostatic sponge.
  b. Tetrode Insertion (Figure 3D).
    i. Slowly lower the tetrodes into the brain at a rate of 10 μm/s until reaching the start of the region of interest (e.g., IPN).
    ii. Once at the initial depth, wait at least 30 min to allow tissue to stabilize and reduce acute insertion effects.
    iii. For final approach to the target region, decrease the descent speed to 1–2 μm/s.
      **CRITICAL:** Slow approach and waiting period are essential to minimize tissue damage and improve recording stability.
  c. Continuously monitor all channels for neuronal spikes using the acquisition software.
  d. Adjust tetrode depth as needed to optimize signal amplitude and isolate single-unit activity. Troubleshooting 6.
    **CRITICAL:** The most important factor is achieving a high signal-to-noise ratio and stable spike amplitudes across channels, which is crucial for reliable spike sorting.
  e. Data Acquisition: Acquire data using a 36-channel preamplifier connected to the Neuralynx Digital Lynx 4SX data acquisition system. Record signals using Cheetah software, ensuring all channels are properly referenced and grounded.
    ***Alternatives:*** Several alternative data acquisition systems are available for multi-channel electrophysiology. Platforms such as the open-source OpenEphys,^39^ Intan Technologies RHD recording systems, and OneBox (imec) offer cost-effective solutions that support high channel counts and are compatible with advanced probes like Neuropixels.^40^ These systems provide flexible hardware and software options for large-scale neural recordings and may be preferable for experiments requiring simultaneous monitoring of many neurons.
  f. Quality Control: Check for stable baseline recordings and robust spike activity before proceeding with pharmacological manipulations.
  g. If signal quality is poor (e.g., high noise, unstable amplitudes), adjust grounding, check connections, or reposition electrodes as needed.
    ***Note:*** Always document electrode depth and coordinates for reproducibility. Maintain detailed logs of all adjustments and observations during recording.
13. Nicotine injection:
  a. Begin each recording with a 5-minute baseline.
  b. Inject either saline or nicotine i.v. in pseudo-random order. Troubleshooting 7.
  c. Wait ≥5 minutes after saline and ≥15 minutes after nicotine before next injection.
  d. For dose-response experiments, inject 7.5, 15, 30, or 60 μg/kg nicotine (2–4 injections per mouse) in pseudo-random order.

**CRITICAL:** Allow adequate washout between injections to avoid cumulative effects. Monitor depth of anesthesia regularly; adjust chloral hydrate accordingly.

14. Confirming Tetrode Placement Post-hoc:
  a. To verify accurate tetrode placement after recordings, apply a small amount of a fluorescent dye (such as DiI, DiO, or DiD)—distinct from any dye used with the infusion cannula—directly onto the tetrode tip approximately 5 minutes before insertion into the brain.
  b. Following the completion of recordings, extract the brain and immerse it in 4% paraformaldehyde (PFA) at 4°C for at least one week to allow for thorough fixation.
  c. Subsequent histological processing and fluorescence imaging can then be performed to confirm the precise location of the tetrode tracks (see section 18 for detailed histology procedures).
15. Data Processing and Analysis.
  a. Detect extracellular spikes from raw data using CSC Spike Extractor (Neuralynx).
    ***Note:*** Adjust detection thresholds to optimize sensitivity and minimize noise artifacts. Visual inspection of detected events is recommended.
  b. Perform spike sorting using SpikeSort3D (Neuralynx), applying manual cluster cutting to isolate single units.
    **CRITICAL:** Carefully inspect clusters for separation and stability. Only include well-isolated units for further analysis.
    ***Note:*** There are many valid approaches to spike sorting; the workflow described here matches Jehl et al.,^1^ but alternative open-source semi-automatic pipelines (Kilosort4^41^ + Phy2, SpikeInterface,^42^ CellExplorer,^43^ etc.) are equally valid depending on your setup and needs. These are more efficient for high-density silicon probes (e.g., Neuropixels) or larger datasets.
  c. For each well-clustered unit, extract:
    i. Spike timestamps (precise timing of each detected spike).
    ii. Waveform data (average or all waveforms for each unit; sometimes referred to as “waveform shapes” or “waveform matrices”).
      ***Note:*** Consistent extraction and storage of spike times and waveforms are essential for downstream analyses.
  d. Data analysis:
    i. Neurons are classified as activated if their firing rate increases after nicotine administration, and as inhibited if their firing rate decreases.
    ii. To determine responsiveness, a bootstrap analysis is performed by shuffling each neuron’s baseline spike intervals 1,000 times to generate a distribution of expected responses.
    iii. The actual nicotine-induced change is then compared to this distribution: a neuron is considered responsive if its change in firing rate falls at or above the 98th percentile (activation) or at or below the 2nd percentile (inhibition) of the shuffled data.
    iv. For quantification, the maximal increase (for activated neurons) or minimal decrease (for inhibited neurons) in firing rate is extracted within the first 3 minutes after injection, and responses to nicotine are compared to those observed after saline administration.

### *In vivo* use of CATCH with behavior

**Timing: 2 weeks**

**Timing: 5–7 days for step 16**

**Timing: 1 h per mouse for step 17**

**Timing: 5 days for step 18**

**Figure 4:**
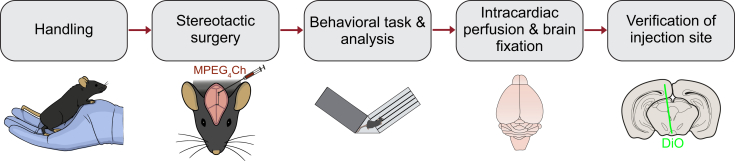
Experimental workflow for the *in vivo* use of CATCH with behavior

This section describes how to apply the CATCH protocol to investigate the functional consequences of sustained, localized receptor antagonism on animal behavior. The following procedures detail the steps for animal handling and habituation, stereotaxic infusion of MPEG_4_Ch, post-surgical care, and behavioral testing, providing a comprehensive framework for linking molecular and cellular manipulations to complex behavioral outcomes (Figure 4). This approach was used in Jehl et al.^1^ to reveal the role of β4 nAChR subunits in nicotine reward, but can be adapted to address a wide range of neuropharmacological questions.

***Note:*** By combining precise chemogenetic manipulation with established behavioral paradigms, such as conditioned place preference (CPP), researchers can directly assess how targeted inhibition of nAChRs in specific brain regions influences reward-related and motivational processes *in vivo*.

16. Handling and weight measurements. This procedure ensures animals habituate to the experimenter and the testing environment, minimizing stress-induced variability in behavioral outcomes.
  a. Transport animals in their home cages to the behavioral testing room, or its antechamber, a few days before the surgical procedure.
  b. Scoop mouse out of cage.
    i. Let the mouse explore your hands and arms for 8–10 minutes.
    ii. Periodically touch the mouse head and back to habituate the mouse to experimenter contact.
    iii. Weight the mouse and return back to the cage.
  c. Repeat procedure for all mice of the batch, changing gloves between cages/when you start handling a different cage.
  d. Repeat handling session once a day for 5 days.

***Note:*** If needed, handling sessions can be increased to 7 days.

***Note:*** In case the behavioral experiment includes an i.p. injection or connecting the animal to an optic fiber, you can habituate it to these procedures in the two last days of handling period. For example, in Jehl et al.,^1^ the last 2 handling sessions comprised 8 min of normal handling and 2 min for one i.p. injection of filtered saline.

**CRITICAL:** To reduce stress-related variability, maintain consistency between the habituation routine and the actual testing procedure. During the behavioral experiment, use the same lab coat wore during the handling phase. Troubleshooting 8.

***Note:*** Conduct behavioral tests within the same fixed time window each day.

17. Preparation and infusion of MPEG_4_Ch for *in vivo* use. The objective is to prepare and infuse MPEG_4_Ch into a target region for subsequent *in vivo* behavioral experiments.
  a. Prior to beginning the procedure, prepare MPEG_4_Ch for local injection as in step 7.
    **CRITICAL:** Prepared MPEG_4_Ch solution should be used within the same day. Prepare a fresh dilution daily before use.
  b. Pull injection glass micropipettes (Drummond 10 μL PCR Pipettes, Cat# 5-000-1001-X10) using a 2-step pull on an upright puller (Narishige PC-10) with the following parameters: Load weight: 250 g, Step 1 heat: 59.6°C, Step 2 heat: 44.9°C, Pull length: 6 mm.
    ***Note:*** Store pulled capillaries in a covered plate to prevent contamination. Minimize air exposure to maintain sterility. Always handle micropipettes with clean gloves.
  c. Prepare for surgery by gathering all the supplies listed in the materials and equipment section and setting up the stereotaxic apparatus (Figure 5).
    i. Sterilize all tools in an autoclave and store them in a sterile container.
    ii. Check that the isoflurane source is full and disinfect all surfaces, including the stereotaxic device.
    iii. Ensure all reagents are available before surgery.
    iv. Ensure that your mouse is at the appropriate age and weigh the mouse to determine the dosage of pre-anesthetic drugs.

**Figure 5:**
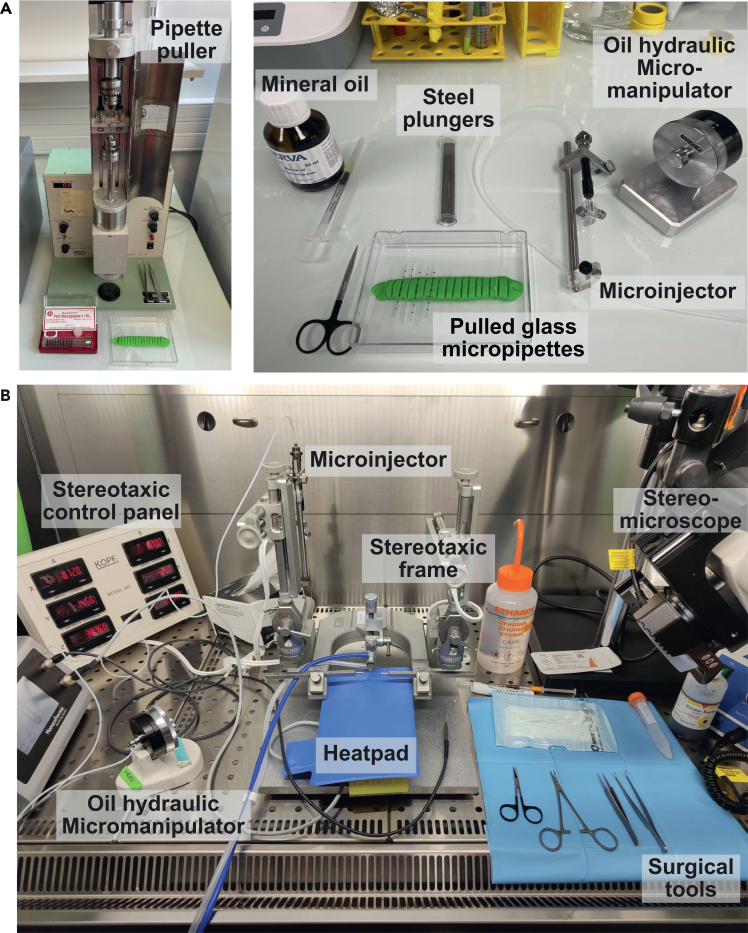
Stereotactic surgery setup (A) An upright micropipette puller, used with thin-walled glass capillaries to produce glass pipettes suitable for intracranial injections. (B) Example of a typical stereotaxic setup, including surgical tools, a stereotaxic frame with ear bars and a nose holder for head immobilization and gas anesthesia, and a control panel for coordinate recording. The oil hydraulic micromanipulator is attached to a manipulator arm to ensures precise intracranial injections. A heat pad maintains body temperature under anesthesia, while a stereomicroscope provides magnified visualization of the surgical field.
  d. Prepare the injection micropipette.
    i. Cut the tip of the glass micropipette with sharp surgical scissors.
    ii. Add Vaseline around the plunger.
    iii. Fill the glass micropipette with mineral oil.
    iv. Insert the plunger into the rear of the micropipette and ensure smooth ejection of mineral oil.
    v. Attach the One-axis Oil Hydraulic Micromanipulator (MO-10, Narishige, Tokyo, Japan) to the stereotaxic arm and insert the micropipette.
    vi. Ensure the plunger moves properly and fluid can be dispensed.
    vii. Load the micropipette with MPEG_4_Ch by touching the fluid bead on parafilm or directly from an Eppendorf tube (with risk of breaking the tip).
    viii. To confirm accurate targeting, mix the MPEG_4_Ch solution with AAV5-GFP virus (use a strong ubiquitous promoter for proper expression; for the IPN, AAV5 at a titer of 5.7 × 10^11^ vg/mL is effective), or a fluorescent dye (DiI, DiO, DiD at the stock concentration of 1 mg/mL).
      ***Alternatives:*** As an alternative you can use a 1 μL of dye and brush the micropipette tip 5 min before entering the brain to check the micropipette track.
    ix. Post hoc microscopic confirmation is required for injection site verification (see step 19).
  e. Induction of anesthesia.
    i. Place the mouse in an induction chamber and administer isoflurane at 3% with 1 L/min Oxygen, regulated with an anesthesia station (MiniHUB V3, TEMSEGA).
    ii. Observe for deep anesthesia before transferring the mouse to the surgical platform.
    iii. Shave the top of the head in two passes: first in the direction of hair growth, then against it.
    iv. Remove all loose hair.
    v. Secure the mouse in the stereotaxic frame by gently pulling the tongue forward.
    vi. Position the incisor bar and connect the respiratory tube.
      ***Alternatives:*** Other anesthetics, such as ketamine/xylazine mix, can be used in place of isoflurane.
  f. Maintain anesthesia with isoflurane at 1.5% with 0.5 L/min Oxygen.
    ***Note:*** If the mouse starts gasping, reduce isoflurane concentration.
  g. Maintain body temperature with a heating mat at 37°C.
  h. Surgical Preparation.
    i. Inject Lidocaine (100 μL) under the skull’s skin at the incision site.
    ii. Administer Buprenorphine (1 mg/kg) 30 min before the end of surgery.
    iii. Apply physiological gel to the eyes to prevent dryness.
  i. Mark the mouse for identification: Left ear → Mouse 1, Right ear → Mouse 2, Both ears → Mouse 3, etc.
  j. Disinfect the incision site with betadine and dry with a sterile cotton bud.
  k. Stereotactic surgical preparation.
    i. Make a midline incision from the eyes to the skull base with a fine scissor.
    ii. Remove conjunctival tissue from the skull surface.
    iii. Identify bregma and lambda as stereotaxic reference points.
    iv. Ensure the skull is level with bregma and lambda before proceeding. If these are <0.05 mm different, you can proceed. If it is >0.05 mm different, adjust the mouse’s placement in the ear bars or the height of the bite bar until the skull is flat in the AP axis.
      ***Note:*** AP distance between bregma and lambda should be around 4.2 mm in an adult C57 mouse.
  l. Mark the target site using stereotaxic coordinates.
    ***Note:*** For the IPN we used the following target: AP: +3.4 mm, ML: ±0.4 mm. Angle: 5°.
  m. Drill through the skull at the marked site using a 0.9 mm steel drill bit, avoiding dura damage.
  n. Injection.
    i. To verify flow, gently expel a small drop of liquid from the micropipette tip. If the pipette appears clogged, moisten the tip with filtered saline and try again.
      ***Alternatives:*** Alternatively, carefully shorten or break the tip using forceps to restore flow. If the issue persists, replace the micropipette.
    ii. Lower the micropipette to the target depth (DV axis).
    iii. Wait 2 min before injecting 500 nL at 100 nL/min (5 full turns on the One-axis Oil Hydraulic micromanipulator).
    iv. After injection wait 4 min, withdraw the needle by 0.1 mm, and wait another 1 min before slowly withdrawing the needle while monitoring for fluid leakage. (Total waiting time = 5 min).
    v. Verify pipette flow after the injection to confirm successful delivery.
  o. Fixation and Closure.
    i. Rehydrate the skull with saline (do not dry or remove excess).
    ii. If the procedure is lengthy, inject 0.9% saline subcutaneously.
    iii. Suture the scalp using 3–4 stitches.
    iv. Clean the incision area with betadine.
  p. Turn off the isoflurane and remove ear bars carefully.
  q. Post-Surgical Care.
    i. Place the mouse on a heating pad for recovery.
    ii. Monitor the mouse until fully awake.
    iii. Check for signs of distress or complications.
    iv. Ensure proper hydration and administer analgesics as per protocol.
    v. Verify carbon filter weight and isoflurane levels before ending the session.
    vi. Monitor mouse during the three days following surgery.
18. Behavioral task: Conditioned Place Preference (CPP) test.
  ***Note:*** The following step describes the conditioned place preference (CPP) task that we performed in Jehl et al.^1^ We used this to investigate the role of β4 nAChR subunits in the regulation of nicotine reward. However, the protocol can be adjusted for other behavioral tests.
  **CRITICAL:** In Jehl et al.,^1^ the CPP test in KI mice was initiated 3 days after MPEG_4_Ch infusion into the IPN. We recommend allowing a 3-day interval between infusion and behavioral testing. However, *ex vivo* data from the IPN indicate that MPEG_4_Ch-mediated antagonism persists for at least 10 days post infusion. This suggests that a longer interval may be possible but should be empirically validated.
  ***Note:*** To minimize stress, avoid changing the cages of experimental animals during the same week as behavioral testing.
  a. Prepare the behavioral testing environment by arranging all necessary equipment including:
    i. Dimmable panel light.
    ii. CPP chamber.
      ***Note:*** We used a Y-maze apparatus (Imetronic, Pessac, France) with one arm closed and the two remaining arms fitted with manually operated doors. This setup created two rectangular chambers (11 × 25 cm), each featuring distinct cues in texture and color, separated by a central triangular neutral compartment (11 cm per side). One chamber had gray textured floors and walls, while the other had smooth floors and walls with black and white stripes.
      **CRITICAL:** The combination of textures and colors chosen for each chamber were balanced in previous pilot experiments to minimize mice’s initial preferences. Troubleshooting 9.
    iii. An overhead video camera (Intel RealSense, 640x480, 30 frames per s).
    iv. A computer with recording software for video acquisition.
      **CRITICAL:** Light intensity during testing significantly influences behavioral outcomes. In this protocol, light levels were maintained at 100 lux within the CPP chambers.
      **CRITICAL:** Animals should be transferred to the testing room or holding area at least 30 min prior to each behavioral session (from Day 1 to Day 5) to allow acclimatization.
  b. Pretest, day 1:
    i. Take each mouse individually and place it in the middle chamber of the CPP apparatus.
      ***Note:*** In this pretest session, allow the mouse to freely explore and access both open arms of the Y maze.
    ii. Record with the overhead video camera the activity of the animal for 15 minutes.
    iii. After placing the mouse back in its cage, thoroughly clean the apparatus using Surfa’Safe Premium disinfectant spray (Laboratoires Anios, #20003N). Change gloves before handling the next mouse, and repeat this process for all animals.
    iv. After the pretest session, select a video analysis method to detect the animal’s position and calculate its trajectories and time spent in each chamber.
      ***Note:*** In Jehl et al.,^1^ we used a custom-made Python recording software to record the video, and ezTrack^34^ to track the position of the animal offline. Alternatively, open-source Bonsai,^44^^,^^45^ or EthoVision and ANY-maze software can be used for behavioral tracking.
    v. Once the time spent in the two chambers is determined, randomly assign individual mice into two balanced groups. In one group, nicotine is paired with one chamber, and in the other group, nicotine is paired with the opposite chamber to ensure an unbiased CPP design.
  c. Conditioning days, day 2 to 4:
    i. Prepare filtered saline (0.9% NaCl) and nicotine tartrate solution (0.2 mg/kg, in filtered saline) as described in materials and equipment section.
      ***Note:*** Each conditioning day includes two conditioning sessions, at least 5 h apart from each other. In one conditioning session an i.p. injection of nicotine is paired with one chamber, whereas in the other session a saline injection is paired with the other chamber. The order of saline - nicotine session is counterbalanced across the 3 conditioning days.
      **CRITICAL:** A saline-saline control group, in which both chambers are paired with saline should be added as a control.
    ii. Scoop mouse out of its home-cage and perform an i.p. injection of either saline or nicotine solution.
    iii. Immediately after injection, place the animal into the appropriate chamber for 20 minutes (videorecording these sessions is not necessary).
    iv. After placing the mouse back in its cage, thoroughly clean the apparatus with a disinfectant spray. Change gloves before handling the next animal, and repeat this process for all mice.
    v. Repeat [c.ii, c.iii, c.iv] for all conditioning sessions on all conditioning days.
  d. Test day, day 5: repeat procedure of pretest [b.i–iv].
  e. Behavioral analysis: The preference score (in seconds) is calculated by subtracting the pretest difference from the test difference using the following formula:
    (tNIC−tSAL)TEST−(tNIC−tSAL)PRE
    where:
    tNIC = time spent in the nicotine-paired chamber.
    tSAL = time spent in the saline-paired chamber.
    TEST = values recorded on the test day.
    PRE = values recorded on the pretest day.
    This formula quantifies the change in preference for the nicotine-paired chamber from pretest to test. Troubleshooting 10 and 11.
19. Post-hoc verification of *in vivo* injection and recording sites.
  a. Perform intracardiac perfusion with 1X PBS followed with 4% PFA to fix the brain (must be done inside a chemical fume hood).
  b. Post-fix the brain in 4% PFA at 4°C for at least 3 days.
  c. Section the fixed brain coronally into 50 μm thick slices using a vibratome (Leica VT1000S).
  d. For verification of injection or tetrode placement, directly visualize fluorescent dye (e.g., DiI, DiO, DiD) in brain sections using epifluorescence microscopy. Troubleshooting 12.
  e. If AAV5-GFP virus or other immunolabeling is used, perform immunohistochemistry with an anti-GFP antibody as needed.
  f. Image sections at 10x magnification with an epifluorescence microscope to confirm the location of MPEG_4_Ch infusion or recording sites.
  g. To visualize the slice images, use the FIJI^35^ software. Save the images as TIFF files for long-term storage.
  h. For whole-brain anatomical mapping, use DMC-BrainMap,^36^ an open-source napari plugin, to register injection sites to the Allen Brain Atlas.

## Expected outcomes

**Figure 6:**
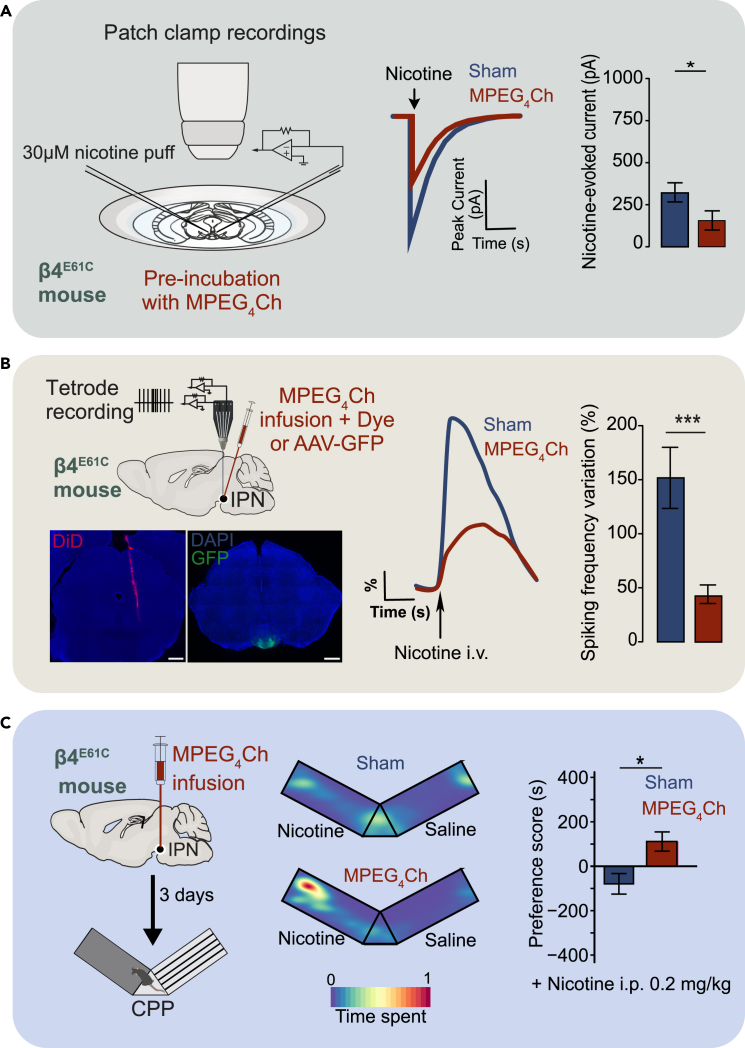
Expected results (A) In *ex vivo* slices, local infusion of MPEG_4_Ch in Chrnb4^E61C^ mice inhibited nicotinic current in IPN neurons. (B) *In vivo* electrophysiological recordings show reduced nicotine-induced activation of IPN neurons after MPEG_4_Ch infusion in Chrnb4^E61C^ mice. Representative immunofluorescence image showing an injection track labeled with fluorescent dye (left), and GFP expression confirming correct targeting in the IPN (right). Scale bars: 500 μm. (C) In a conditioned place preference (CPP) test, blockade of β4 nAChR subunits in the IPN via MPEG_4_Ch infusion increases preference for a low nicotine dose (0.2 mg/kg i.p) in Chrnb4^E61C^ mice. Data are mean ± SEM. ∗*p* < 0.05 and ∗∗*p* < 0.001. Figure reprinted and adapted with permission from Jehl et al., 2025.^1^

When performed as described, this protocol enables robust and reproducible assessment of CATCH-mediated receptor antagonism at multiple levels. *Ex vivo*, acute brain slice recordings from Chrnb4^E61C^ KI mice treated with MPEG_4_Ch (50 μM, 10 min) show a pronounced and sustained reduction in nicotine-evoked whole-cell currents. In Jehl et al.,^1^ MPEG_4_Ch-treated slices exhibited an average reduction of over 80% in peak nicotine-induced current amplitude compared to untreated controls, confirming the efficacy and specificity of the approach (Figure 6A). This antagonism was selective for the targeted nAChR subunit and persisted for the duration of the recording, while untreated slices maintained stable, robust responses.

*In vivo*, local infusion of MPEG_4_Ch results in sustained antagonism of nAChRs, as evidenced by altered neuronal firing patterns in electrophysiological recordings. Neurons in the target region display either reduced or abolished nicotine - induced activation, and these changes can be quantitatively analyzed using the described spike sorting and statistical methods (Figure 6B). At the behavioral level, animals subjected to CATCH manipulation exhibit altered responses in paradigms such as conditioned place preference (CPP), reflecting the functional consequences of localized, sustained receptor blockade (Figure 6C).

Together, these outcomes provide a comprehensive demonstration of the specificity, efficacy, and functional impact of the CATCH approach, serving as a benchmark for users to evaluate the success of their own experiments.

## Limitations

While this protocol offers powerful and precise control over receptor function, several limitations should be considered.

This protocol is optimized for use with the Chrnb4^E61C^ KI mouse line, which ensures selective and stable expression of the engineered receptor subunit throughout development and across relevant brain regions. While this genetic approach provides high specificity and reproducibility, it does limit the method’s immediate applicability to other receptor types or species unless analogous KI lines are available. Generating new KI lines can be time-consuming and resource-intensive.

As an alternative, viral vectors can be used to express cysteine-mutated receptors in specific brain regions or cell types; however, this approach may result in incomplete replacement of native subunits and variable expression levels, potentially reducing the precision and consistency of receptor targeting.

## Troubleshooting

### Problem 1

Unhealthy brain slices.

In whole-cell patch-clamp recordings (step 3), slice health is critical for stable and reliable data. Unhealthy or deteriorating slices may result from poor slicing conditions, excessive delay after dissection, or inadequate maintenance of physiological conditions during the experiment. This can lead to unstable recordings or failure to obtain usable data.

### Potential solution

• Perform transcardiac perfusion efficiently to minimize ischemia, and keep the cutting solution ice-cold and well-oxygenated throughout dissection and slicing.
• Record only from slices within the first 6 hours after dissection, when tissue viability is highest.
• Continuously bubble all solutions, including MPEG_4_Ch-containing 1X aCSF, with carbogen gas to prevent hypoxia.
• If issues persist, thoroughly clean all experimental materials (slicing tools, chambers, tubing, reservoirs, perfusion lines) and re-prepare all solutions to eliminate possible contamination or degradation.

### Problem 2

No response to nicotine puffs (step 4).

Possible causes include a clogged puff pipette, low nicotine concentration, or failure to deliver the solution.

### Potential solution

Test puff delivery outside the slice before recording. If blocked, refill and clean the lines with filtered 1x aCSF or ethanol followed by Milli-Q water. Check the pneumatic pump pressure. If the problem persists, prepare a fresh nicotine solution.

### Problem 3

No change in nicotine-evoked current amplitude after MPEG_4_Ch incubation (step 5).

Possible causes include lack of receptor expression in the recorded neurons, degraded MPEG_4_Ch aliquots, incorrect stock concentration or dilution errors.

### Potential solution

• Verify receptor expression in the target areas or cells using available assays, such as immunostaining.
• Include positive control experiments in brain regions with confirmed receptor expression.
• Assess MPEG_4_Ch integrity using high-performance liquid chromatography (HPLC), optionally coupled with mass spectrometry.
• Use freshly prepared aliquots and dilute only into freshly oxygenated 1x aCSF immediately before use. Re-prepare the working or stock solution if needed.

### Problem 4

Difficulty with saphenous vein catheterization (vein collapse, poor visualization, bleeding, swelling, or unsuccessful insertion; step 11).

Possible causes include dehydration, vasoconstriction, insufficient exposure, accidental puncture through both sides of the vein, rough handling, or the needle not being in the vein lumen.

### Potential solution

• Warm the limb to dilate the vein and ensure the animal is well-hydrated.
• Carefully dissect and fully expose the vein; use a stereomicroscope if needed.
• Use gentle, shallow insertion to avoid puncturing through the vein.
• If bleeding or swelling occurs, apply gentle pressure with a cotton swab and try a new site along the vein or switch to the other limb.
• If saline does not flow freely, withdraw slightly and reinsert the needle, check for kinks or blockages in the tubing, and flush tubing before insertion.

### Problem 5

Catheter instability, leakage, or loss of patency during the experiment (step 11).

Possible causes include inadequate fixation, animal movement, glue not set, poor seal, or excessive movement/tension on the tubing.

### Potential solution

Hold the needle steady until the glue sets and use only a minimal amount to avoid occlusion.

Ensure the animal is immobilized and the tubing is not under tension.

Reapply glue if leakage occurs and check fixation before proceeding.

Minimize movement during the experiment to maintain catheter patency.

### Problem 6

No or weak signal high noise, or unstable baseline during *in vivo* electrophysiology (step 12).

Possible causes include poor electrode placement, blocked or damaged tetrodes, tissue damage, poor grounding, electrical interference, anesthesia fluctuations, animal movement, electrode drift, or tissue swelling.

### Potential solution

• Verify stereotaxic coordinates and carefully check tetrode integrity before insertion; reposition the probe if needed and allow tissue to settle after insertion.
• Ensure all ground and reference connections are secure; minimize cable and animal movement; shield the setup from external electrical noise.
• Monitor and maintain stable anesthesia throughout the recording.

### Problem 7

Nicotine response is absent or blunted in both control and MPEG_4_Ch conditions (step 13).

Possible causes include incorrect catheter placement, degraded nicotine solution, or anesthesia depth suppressing neuronal responsiveness.

### Potential solution

• Verify intravenous catheter patency before each injection.
• Prepare fresh nicotine solution and confirm correct dosing.
• Monitor anesthesia depth and adjust as needed to maintain physiological responsiveness.

### Problem 8

Mice remain anxious or show high stress during handling or testing (step 16).

Possible causes include inconsistent handling routine, insufficient habituation, handling by multiple experimenters, or sudden environmental changes.

### Potential solution

Ensure handling is performed daily at the same time, by the same experimenter, and in a calm environment. Increase the number of habituation days if needed. Minimize noise and disturbances. Use the same lab coat and gloves as during testing.

### Problem 9

All mice prefer one chamber in the pretest (CPP; step 18).

Possible causes include unbalanced cues (texture, color, light) or strong innate preference.

### Potential solution

Pilot test chamber cues and balance them across groups. Adjust features such as color and texture combinations to minimize innate preference. Randomize the assignment of drug-paired chambers for each animal.

### Problem 10

No behavioral effect of CATCH manipulation (step 18e).

Possible causes include ineffective infusion (missed target, degraded compound), insufficient receptor blockade, or absence of the target receptor.

### Potential solution

• Confirm infusion site with histology.
• Use freshly prepared MPEG_4_Ch solution.
• Confirm receptor targeting and blockade with *ex vivo* or *in vivo* electrophysiology in a subset of animals.
• Consider increasing the MPEG_4_Ch concentration or adjusting the timing between infusion and behavioral testing.
• Include positive control experiments targeting brain regions with confirmed receptor expression.

### Problem 11

Off-target effects or unexpected behavioral/physiological changes after MPEG_4_Ch infusion (step 18e).

Possible causes include diffusion of MPEG_4_Ch outside the intended region, excessive volume, or non-specific binding.

### Potential solution

• Reduce infusion volume or slow the infusion rate to minimize spread.
• Use a fluorescent dye to visualize the extent of spread post-hoc and adjust parameters accordingly.
• Confirm that the observed effects are absent in wild-type or non-targeted animals to rule out non-specific actions.
• Consider using a control ligand or vehicle infusion as an additional control.

### Problem 12

No dye or marker detected at the intended site during histology (step 19).

Possible causes include dye washed out during processing, incorrect dye used, incorrect loading of mix of MPEG_4_Ch, clogging of the pipette, or insufficient fixation.

### Potential solution

Use appropriate dye concentration and fixation protocol. Check carefully when loading solution in pipette and check flow of solution in pipette right before lowering it to inject. Solution should be vortexed right before loading of the pipette to preserve its homogeneity. Minimize time between brain extraction and fixation. Use a dye distinct from other markers. Protect dye from light.

## Resource availability

### Lead contact

Further information should be directed to and will be fulfilled by the lead contact, Nicolas Guyon (nicolas.guyon@espci.fr).

### Technical contact

Technical questions on executing this protocol and requests for resources and reagents should be directed to and will be answered by the technical contact, Alexandre Mourot (alexandre.mourot@espci.fr).

### Materials availability

The KI mouse line Chrnb4^E61C^ and the covalent antagonist MPEG_4_Ch generated in this study will be made available on request, but we may require a completed materials transfer agreement. Additionally, MPEG_4_Ch is commercially available at Broadpharm (San Diego, CA, USA).

### Data and code availability

This study did not report datasets or original code.

## Acknowledgments

The authors would like to thank Joachim Jehl, Eléonore Vicq, Hélène Geoffroy, as well as Otilia de Oliveira and Emilie Tubeuf at the animal facility at ESPCI Paris, for their assistance. We thank DIM C-BRAINS (Région Ile-de-France) and NeuroPSL (Université PSL) for their support. Images of beakers^46^ and mouse^47^ in graphical abstract and of brain^48^ in Figure 4 were adapted from SciDraw (https://scidraw.io/).

This work was funded by the Agence Nationale de la Recherche (ANR-21-CE16-0012 CHOLHAB to A.M., ANR-21-CE37-0026 NICOPTOTOUCH to A.M., and ANR-24-CE16-6957 NIMONAR to A.M.), the Human Frontier Science Program (RGP0035/2020 to A.M.); the National Institutes of Health (NS111600 to A.M.), the Fondation pour la Recherche Médicale (Grant Equation 201903007961 to A.M. and a fourth-year PhD fellowship FDT202404018105 to M.C.), and a Swedish Research Council Post-doctoral fellowship (2022-06168 to N.G.).

## Author contributions

A.M. and N.G. conceptualized and supervised the protocol. A.M. provided the resources. M.C., K.S., A.M., and N.G. contributed to writing the original draft and were involved in writing, reviewing, and editing the manuscript. M.C., K.S., A.M., and N.G. contributed to the visualization. A.M. and N.G. acquired the funding.

## Declaration of interests

The authors declare no competing interests.

## Declaration of generative AI and AI-assisted technologies in the writing process

During the preparation of this work, the authors used Sana AI (Sana Labs) and ChatGPT in order to improve language and readability, with caution. After using this tool, the authors reviewed and edited the content as needed and take full responsibility for the content of the published article.
